# Diversity of clinical phenotypes in a cohort of Han Chinese patients with *PAX6* variants

**DOI:** 10.3389/fgene.2023.1011060

**Published:** 2023-02-02

**Authors:** Lijuan Huang, Jiajia Peng, Yan Xie, Yunyu Zhou, Xiaolin Wang, Hui Wang, Jingang Gui, Ningdong Li

**Affiliations:** ^1^ Department of Ophthalmology, The Second Affiliated Hospital of Fujian Medical University, Quanzhou, China; ^2^ Department of Ophthalmology, Beijing Children’s Hospital, Capital Medical University, Beijing, China; ^3^ Laboratory of Tumor Immunology, Beijing Children’s Hospital, National Center for Children’s Health, Beijing Pediatric Research Institute, Capital Medical University, Beijing, China; ^4^ Department of Ophthalmology, Shanghai General Hospital, Shanghai, China; ^5^ Department of Ophthalmology, Children’s Hospital, Capital Institute of Pediatrics, Beijing, China

**Keywords:** aniridia, *PAX6* gene, mutation, phenotype, minigene

## Abstract

The *PAX6* gene plays an important role in ocular development. Mutations of the *PAX6* gene may result in a series of ocular abnormalities, including congenital aniridia, anterior segment dysgenesis (ASD), progressive corneal opacification, glaucoma, and hypoplasia of the fovea and optic nerve, leading to reduced visual acuity and even blindness. This study aimed to describe the diversity of clinical features caused by *PAX6* pathogenic variants in 45 Han Chinese patients from 23 unrelated families. All patients underwent detailed clinical assessment. Genetic testing was performed to identify pathogenic variations in the *PAX6* gene by next-generation sequencing, minigene splicing assay, RT-qPCR, and long-range PCR. Twenty pathogenic variations were detected in the *PAX6* gene from 12 pedigrees and 11 sporadic patients, of which 12 were previously reported and 8 were novel. The clinical phenotypes obtained as a result of the *PAX6* gene mutations were complicated and vary among patients, even among those who carried the same variants. Genetic testing is helpful for differential diagnosis. Our genetic findings will expand the spectrum of pathogenic variations in the *PAX6* gene. *PAX6* pathogenic variants not only cause defects in ocular tissues, such as the iris and retina, but also lead to maldevelopment of the whole eye, resulting in microphthalmia.

## 1 Introduction

The *paired box 6* (*PAX6*) gene is located on chromosome 11p13 and encodes a 422-amino-acid protein that consists of a conserved paired box domain (PD), a paired homeobox domain (HD), and a proline-, serine-, and threonine (PST)-rich transactivation-enriched domain. *PAX6* regulates the transcription of target genes through its PST domain by binding the DNA to its PD and HD (Prosser and van Heyningen, 1998).

The *PAX6* gene plays an important role in the development of the eye and other organs, such as the nose, pancreas, pituitary gland, and the central nervous system (Simpson and Price, 2002). Mutations of the *PAX6* gene may induce various ocular abnormalities, including congenital aniridia, anterior segment dysgenesis (ASD), cataract, corneal opacification, glaucoma, hypoplasia of the fovea and optic nerve, and nystagmus, leading to reduced visual acuity and even blindness (You et al., 2020).

Congenital aniridia is the most common ocular disorder caused by the *PAX6* gene mutations and is characterized by complete absence of the iris. It has been reported that more than 90% of aniridia cases are caused by the *PAX6* gene mutations, while a few cases are caused by mutations in the *FOXC1*, *PITX2*, *CYP1B1*, *FOXD3*, *ITPR1*, and *TRIM44* genes (Tibrewal et al., 2022).

To date, more than 700 mutations in *PAX6* have been reported before April, 2022, in the Human Gene Mutation Database (https://www.hgmd.cf.ac.uk/ac/index.php), including missense and non-sense mutations, splice-site mutations, insertions, and deletions. Mutations may be distributed in both coding and non-coding regions, such as regulatory enhancer regions. Two-thirds of aniridia cases are caused by loss-of-function mutations in one copy of *PAX6*, and one-third of the cases are caused by chromosomal rearrangements. Clinical phenotypes caused by mutations in *PAX6* have high clinical heterogeneity, depending on the type and location of the mutation. Chromosomal aberrations such as deletions, translocations, and inversions encompassing the complete *PAX6* gene or a part of the *PAX6* gene may lead to isolated aniridia or severe WAGR syndrome, which is characterized by Wilms tumor, aniridia, genital malformations, and intellectual disability due to a large deletion on the 11p13 region encompassing the *PAX6* gene and its adjacent *WT1* gene. Missense mutations may either produce a severe isolated aniridic phenotype or lead to other disorders, such as microphthalmia, microcornea, foveal hypoplasia, ocular coloboma, keratitis, congenital cataract, Gillespie syndrome, Peters anomaly, and morning glory syndrome (Tibrewal et al., 2022). To further understand and expand the spectrum of phenotypes and genotypes of *PAX6*, we analyzed the clinical features of 45 patients with *PAX6* gene variants.

## 2 Materials and methods

### 2.1 Patients

Patients diagnosed with ASD, congenital aniridia, or hypoplasia of the fovea and optic nerve were recruited. All patients underwent ocular examinations, including the best-corrected visual acuity, slit-lamp and ultrasound biomicroscopy (UBM, Guangzhou Sonostar Technologies Co., Limited, Guangzhou, China) for the anterior segment of the eye, ophthalmoscopy and photography for fundus examination, and optical coherence tomography (OCT, Heidelberg Engineering, Heidelberg, Germany) for the retinal structure. Genetic testing was performed for all patients and their family members, following the provision of informed consent, and this study was conducted in accordance with the principles of the Helsinki Declaration and approved by the Ethics Committee of Beijing Children’s Hospital (No. 2016-42).

### 2.2 Genetic testing

Three milliliters of peripheral venous blood was collected from each participant. The genomic DNA was extracted from blood lymphocytes according to the standard protocol (Roche Biochemical, Inc., Palo Alto, CA, United States). A two-step genetic testing strategy was pursued for this cohort, in which next-generation sequencing was followed when no pathogenic or likely pathogenic variants were detected from Sanger sequencing of coding regions in *PAX6*. All potential pathogenic variants (PPVs) identified by NGS were further validated using the Sanger sequencing method. Once the *PAX6* variant was identified, genetic testing was performed on the patients’ family members. Primer pairs used for PCR are listed in Supplementary Table S1.

Next-generation sequencing was performed through a commercial service (MyGenostics Inc., Beijing, China). Briefly, the enriched DNA library was constructed and sequenced on an Illumina HiSeq X Ten platform (Illumina, San Diego, United States). The amplified DNA was captured using the GenCap capture kit (MyGenostics Inc., Beijing, China), which consists of 1,168 eye disease-causing genes that were curated from the Orphanet (https://www.orpha.net) and OMIM databases (https://www.omim.org/). The probes were designed to capture the whole coding and non-coding regions known to be involved in pathogenic variants associated with eye diseases, including the *PAX6* gene related to aniridia. The clean reads (<150 bp) were mapped to the UCSC hg19 human reference genome after removing duplicated and low-quality reads. Variants were analyzed through the Genome Analysis Toolkit (GATK) program, and only variants with a GATK-assigned quality criterion score >50.0 were considered for downstream analyses. Variants were further annotated by ANNOVAR software (http://annovar.openbioinformatics.org/en/latest/), which combines multiple public databases, such as 1000 Genomes, ESP6500, dbSNP, gnomAD, ClinVar, HGMD, and one commercial MyGenostics database. Copy number variants were detected by CNVkit based on the readDepth algorithm (Talevich et al., 2016). The pathogenicity of missense mutations was predicted by PolyPhen-2 (http://genetics.bwh.harvard.edu/pph2/), Sorting Intolerant From Tolerant (SIFT, https://sift.bii.a-star.edu.sg/), and Combined Annotation-Dependent Depletion (CADD) (https://cadd.gs.washington.edu/). The splicing effects of the variants were assessed using the SpliceAI program (Jaganathan et al., 2019) and CADD.

Only candidate variants associated with the clinical features of the patients were retained for inheritance analysis in the core family members available by Sanger sequencing. The inherited variant showing non-segregated phenotypic evidence in the core family was excluded. The pathogenicity of the remaining variant was interpreted, according to the guidelines of the American College of Medical Genetics and Genomics (ACMG) (Richards et al., 2015). A 3D model of the non-synonymous mutant protein was visualized using the PyMOL program (Rigsby and Parker, 2016).

### 2.3 Minigene assay

A minigene splicing assay was conducted to confirm the splicing error caused by the splicing acceptor site variant of c.-128-2A>G in the 5′UTR region of *PAX6.* The target region, including the exons 3 and 4 and the corresponding introns, was amplified with the following primer pair, forward, 5′CGC​TGA​AAA​TGT​GGG​TGT​AAT3′ and reverse, 5′TAT​CGA​GAA​GAG​CCA​AGC​AAA​C3′, with the DNA from the peripheral blood mononuclear cells of a healthy volunteer and the patient used as a template. An amplicon was inserted into the p-EASY-T1 cloning vector, according to the manufacturer’s instruction (TransGen Biotech, Beijing, China).

Cloning sequencing was performed to verify the target sequences. A mixture of pET01 Exontrap (Chen et al., 2018) and p-EASY-T1 cloning vectors was digested with restriction endonucleases of BamHI and NotI. The digested pET01 Exontrap vectors were circularized with the target DNA fragment by self-ligation in a 20-μl reaction mixture containing 2-μl ligation buffer and 2-μl T4 DNA ligase (Promega, Madison, WI, United States) at 4°C for 16 h. The resultant Exontrap plasmid with the *PAX6* minigene was subsequently transfected into HEK293T and COS7 cells using the Lipofectamine 2000 DNA transfection reagent (Invitrogen, Carlsbad, CA, United States). Total RNA was extracted from the cells. The reverse transcription reaction was performed using the PrimeScriptTM RT Master Mix (Dalian TaKaRa Biotechnology, Co., Ltd., Dalian, China). The minigene cDNA was amplified using the pET01 Exontrap plasmid-specific primers with an initial denaturation of 2 min at 94°C, followed by 35 cycles of 30 s at 94°C, 30 s at 58°C, 45 s at 72°C, and a final 10-min extension at 72°C. The PCR products were subsequently validated by Sanger sequencing.

### 2.4 qPCR and long-range PCR

Long-range PCR and qPCR were performed to detect deletions in the *PAX6* gene in Pedigree 8, which showed complex ocular phenotypes. The primer pairs used for qPCR and long-range PCR are listed in Supplementary Table S2. PCR amplification was performed in a 15-μl buffer containing 5-ng DNA, 2-µM of each primer, and 7.5-µl SYBR Green PCR Master Mix (Thermo Fisher Scientific, Waltham, MA, United States). RT-PCR was performed using the TB Green Premix Ex Taq II reagent (RR0360, TaKaRa), according to the manufacturer’s protocol. PCR products were calculated using the comparative Ct method (ΔΔCt), with an equation of 2^-ΔΔCt^. Amplicons from the exon 6 of *SPATA7* and exon 14 of *TTLL5* were used as the positive controls. The primer pairs for the amplification of internal reference genes (*SPATA7* and *TTLL5* genes) and the length of the PCR product are listed in Supplementary Table S3.

**FIGURE 1:**
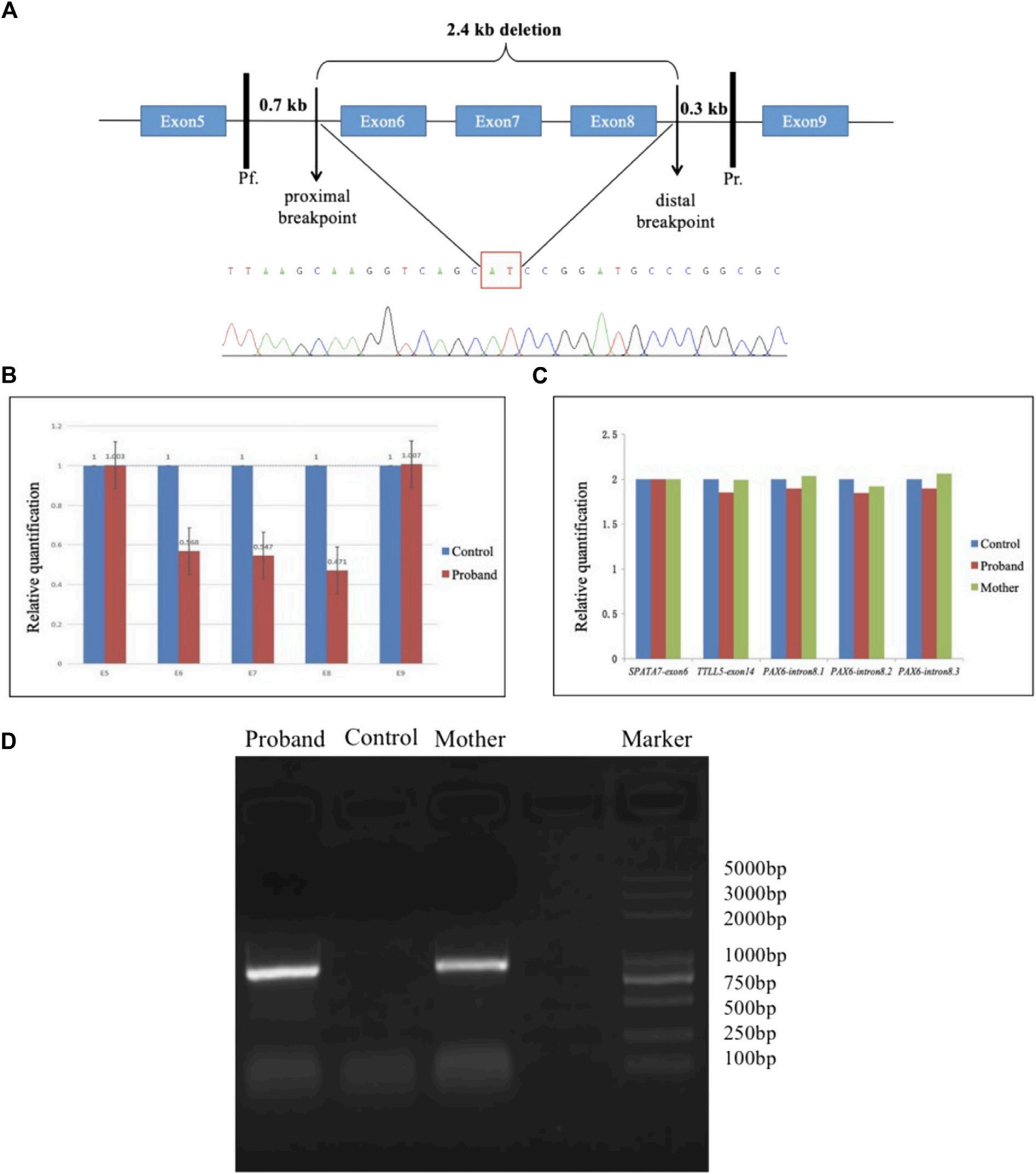
Long-range PCR and sequence analysis. **(A)** Schematic diagram demonstrates a 2.4-kb deletion in the *PAX6* gene, including whole exons 6–8. The site of the primer Pf was located 0.6–0.7 kb upstream of the proximal breakpoint, while the primer Pr was located 0.2–0.3 kb downstream of the distal breakpoint. The *PAX6* exons were denoted with blue boxes. The partial sequences located at the upstream and downstream breakpoints are shown in the cloning sequencing chromatograms with a red rectangle surrounding the breakpoint. (B & C) Histogram indicates the relative quantity (RQ) between the exons 5–9 of *PAX6* and the control. The copy number ratio for the amplicons of exons 6–8 was half-compared to the control **(B)**. In contrast, the copy number for the amplicon of intron 8 of *PAX6* was closed to that approached to those of exon 6 of *SPATA7* or exon 14 of *TTLL5* **(C)**. **(D)** Agarose gel electrophoresis of PCR products with a final 2-min extension condition. An approximate 0.9-kb band was produced from two affected individuals (the proband and his mother in F8) through long-range PCR amplification but not produced from a normal individual due to a short extension time.

Long-range PCR amplification was performed using the following primer pair: Pf, 5′-ACC​CGG​CAG​AAG​ATT​GTA​GAG -3′; Pr, 5′-CCC​AGT​GTC​CGT​CCT​ATA​TTG​T-3′ in a 50-μL mixture buffer containing 5 U of LA Taq polymerase (Dalian TaKaRa Biotechnology, Co., Ltd., Dalian, China), 2.5 mM of MgCl_2_, 0.4 mM of each dNTP, 0.5 μM of each primer, and 50 ng of DNA. The primer Pf was located approximately 0.6–0.7 kb upstream of the proximal breakpoint in exon 5, while the primer Pr was located 0.2–0.3 kb downstream of the distal breakpoint in intron 8 (Figure 1). The resulting PCR product was predicted to be approximately 3.3 kb in a normal individual and was estimated to be approximately 0.9 kb in the patient having a large deletion. The PCR reaction was performed under the conditions of an initial 2-min denaturation at 98°C, followed by 35 cycles of 15 s at 98°C, 20 min at 66°C, and a final 2-min extension step at 72°C. The amplicons were separated by electrophoresis on 1.2% agarose gels and extracted using a QIAquick Gel Extraction Kit (QIAGEN, Valencia, CA, United States). The purified DNA was sequenced and analyzed.

## 3 Results

### 3.1 Clinical assessment

**FIGURE 2:**
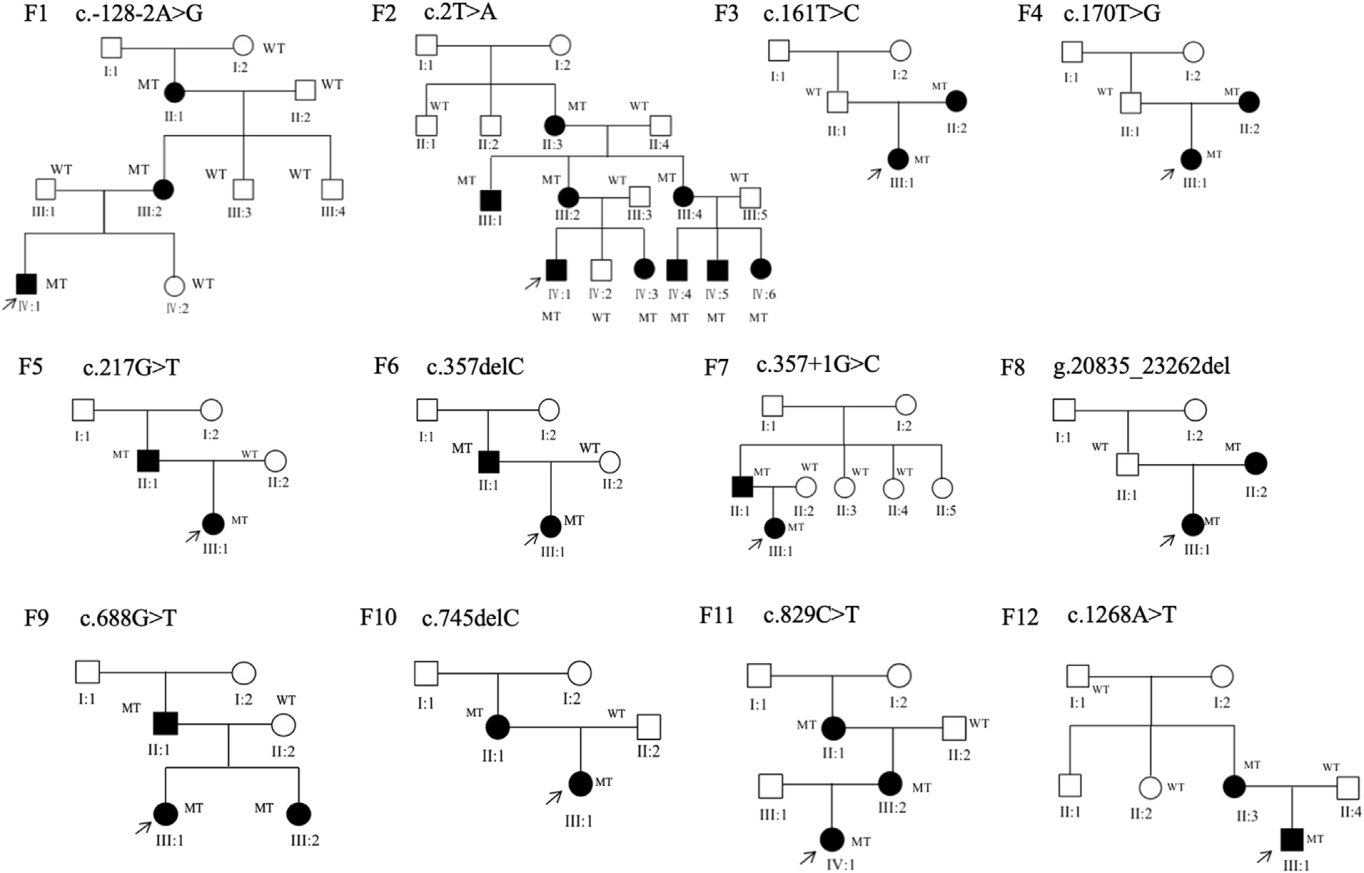
Family structure of 12 families in this study. Squares indicate male individuals, circles indicate female individuals, and solid symbols indicate affected individuals. MT, mutant type; WT, wild type.

**FIGURE 3:**
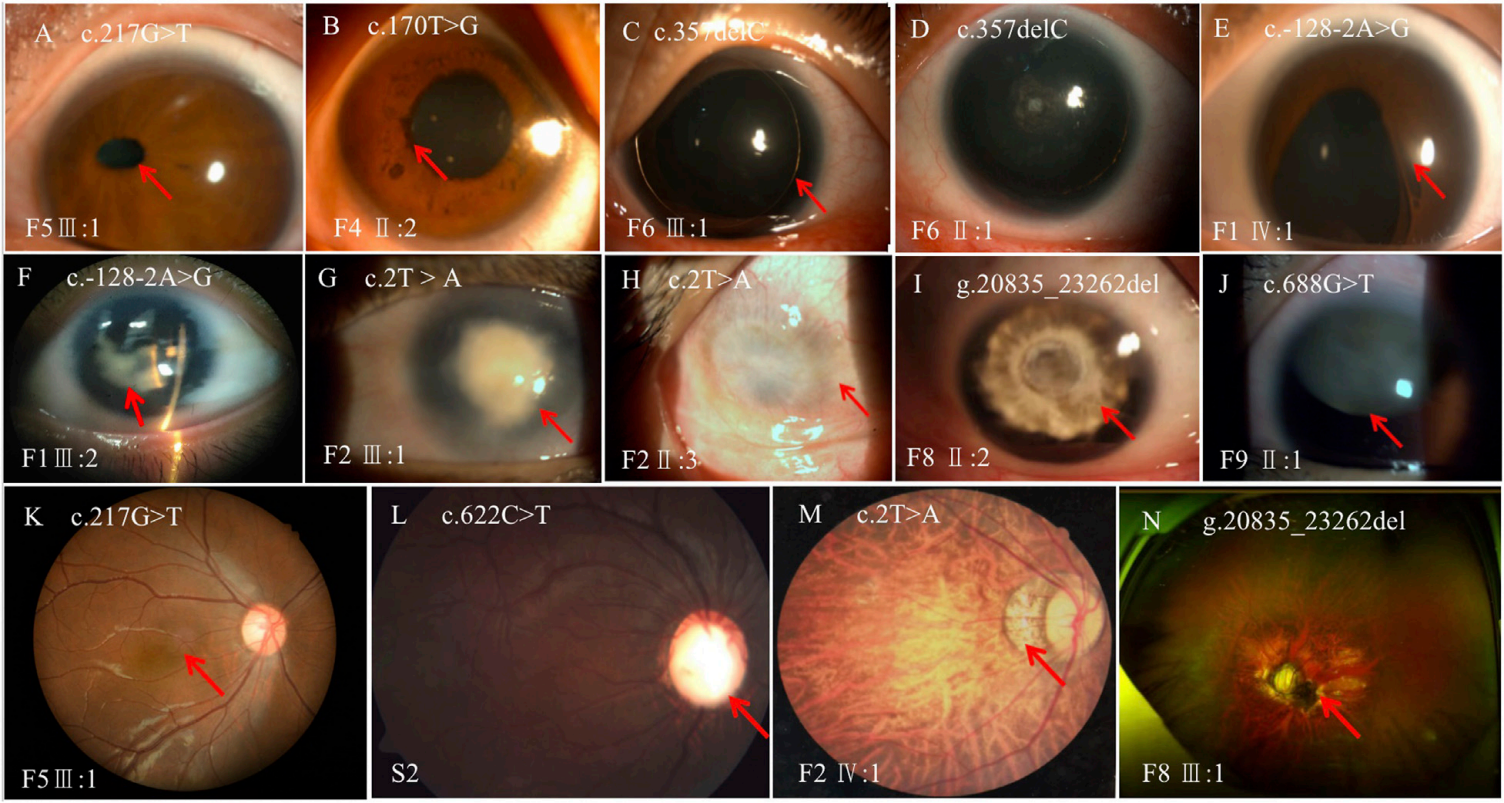
Variable phenotypes of the anterior segment and fundus in patients with *PAX6* variations. **(A)**, ectopic pupil; **(B)**, iris ectropion uvea; **(C)**, complete absence of the iris; **(D)**, complete absence of the iris and mild cataract; **(E)**, partial absence of the iris with an irregular pupil; **(F)**, complete absence of the iris and cataract; **(G)**, keratopathy; **(H)**, corneal vascularization and opacification; **(I)**, aniridia and cataract; **(J)**, aniridia and ectopic lens; **(K)**, foveal hypoplasia; **(L)**, optic nerve atrophy; **(M)**, choroidal coloboma; **(N)**, dysgenesis of the optic disc and nerve. The lesion is labeled with the red arrow.

Thirty-four patients from 12 pedigrees (F1–F12) and 11 sporadic patients were clinically evaluated in this study, including 23 probands. There were nine affected individuals in the F2 pedigree, whereas there were no more than five affected individuals in the remaining 11 pedigrees (F1 and F3–F12) (Figure 2). Eight pedigrees (F1, F4, and F6–F11) were diagnosed with congenital aniridia as many patients in these pedigrees showed a complete absence of the iris, although a few patients in these pedigrees presented with an incomplete absence of the iris. Other clinical findings in these eight pedigrees included nystagmus, cataract, microcornea, ectopic lens, corneal vascularization and opacification, foveal hypoplasia, high myopia, exotropia, and ptosis (Figure 3A–N). A suspected aniridia disorder was diagnosed in two pedigrees (F3 and F12) because the patients in these two pedigrees showed iris coloboma and congenital cataract (Supplementary Figure S1). Congenital foveal hypoplasia was diagnosed in two pedigrees (F4 and F5) where patients did not have evidential iris changes. Ten of 11 sporadic patients were diagnosed with congenital aniridia. One patient was diagnosed with congenital optic nerve hypoplasia and high myopia. In pedigree F1, the proband (a 4-year-old boy), who inherited a pathogenic variation of c.-128-2A>G from his mother (a 34-year-old women), showed a partial absence of the iris (Figure 3E), and his mother presented a complete absence of the iris and cataract (Figure 3F). All patients had reduced visual acuity. The summary of clinical phenotypes of all patients is listed in Table 1. The detailed clinical features are listed in Supplementary Table S4.

**TABLE 1:** Summary of clinical phenotypes of patients in this study.

	Number	Percentage (%)
**Gender**		
Male	20	44.44
Female	25	55.56
**Clinical presentation**		
Iris hypoplasia		
Complete	34	75.56
Partial	5	11.11
Full iris	6	13.33
Foveal hypoplasia	45	100.00
Keratopathy	12	26.67
Microphthalmia	9	20.00
Cataract	8	17.78
Glaucoma	3	6.67
High myopia	3	6.67
Ectopic lens	2	4.44
Strabismus	2	4.44
Ectopic pupil	1	2.22
Optic nerve hypoplasia	1	2.22
Dysgenesis of the optic disc	1	2.22
Choroidal coloboma	1	2.22
Iris ectropion uvea	1	2.22

### 3.2 Genetic findings and functional analysis

Twenty pathogenic variations were detected in the *PAX6* gene of 34 patients from 12 pedigrees and an additional 11 sporadic patients, including 8 novel and 12 previously reported variants (Table 2). These variants were mainly distributed in the domains of PD, LINK, and PST.

**TABLE 2:** Summary of detected pathogenic variations of *PAX6* in this study.

No.	Case	cDNA	Amino acid change	Location	Domain	ACMG	Evidence level	CADD	SIFT	PolyPhen-2	Splice AI	Type of mutation	Reference
1	Familial	c.-128–2 A>G		Intron 2	UTR’5	Pathogenic	PVS1+PM2+PP4	—	—	—	AL (0.65)	Splicing	This study
2	Familial	c.2T>A	p.Met1Lys	Exon 4	PD	Pathogenic	PP1 + PVS1 + PM2 +PS4 +PP4	24.1	D	B	N/E	Missense	PMID: 26535646
3	Sporadic	c.141G>A	p.Gln47Gln	Exon 5	PD	Likely pathogenic	PS2+PM2 +PP3+PS4 +PP4	26.9	—	—	DL (0.73) DG (0.31)	Synonymous	PMID: 25678763
4	Sporadic	c.141 + 2T>C		Intron 5	PD	Pathogenic	PVS1+PS2+PS4 + PM2 +PP4	33	—	—	DL (0.99)	Splicing	PMID: 12782766
5	Familial	c.161T>G	p.Val54Gly	Exon 6	PD	Likely pathogenic	PM5+PM2 +PP4	27.5	D	—	N/E	Missense	This study
6	Familial	c.170T>A	p.Leu57Gln	Exon 6	PD	Pathogenic	PM2 +PP3+PP4	28	D	P	N/E	Missense	This study
7	Familial	c.217G>T	p.Gly73Cys	Exon 6	PD	Likely pathogenic	PM2 +PP3+PP4	29.8	D	P	N/E	Missense	This study
8	Familial	c.357delC	p.Ser119Argfs*5	Exon 6	PD	Likely pathogenic	PVS1+PM2+PP4	—	—	—	-	Deletion	This study
9	Familial	c.357 + 1G>C		Intron 6	PD	Pathogenic	PVS1+PS4 +PM2 + PP4	33	—	—	DL (0.99)、DG (0.5)	Splicing	PMID: 9452088
10	Familial	g.20835_23262del		Exons 6–8	PD + HD	Pathogenic	PVS1+PM2+PP4	—	—	—	—	Deletion	This study
11	Sporadic	c.468G>A	p.Trp156*	Exon 8	LNK	Pathogenic	PVS1+PS2+PM2 + PP4	—	—	—	—	Non-sense	PMID: 25525159
12	Sporadic	c.575_576del	p.Ser192fs	Exon 8	LINK	Pathogenic	PVS1+PS4 +PM2 + PP4	—	—	—	—	Deletion	This study
13	Sporadic	c.613C>T	p.Gln205*	Exon 8	LNK	Pathogenic	PVS1+PS2+PS4 + PM2 +PP4	—	—	—	—	Non-sense	PMID: 12721955
14	Sporadic	c.622C>T	p.Arg208Trp	Exon 8	LINK	Likely pathogenic	PS2+PS4+ PP1+PM2 +PP3+PP4	29.4	D	P	N/E	Missense	PMID: 8364574
15	Familial	c.688G>T	p.Glu230*	Exon 9	HD	Pathogenic	PVS1+PP1+PM2 + PP4	—	—	—	—	Non-sense	This study
16	Familial	c.745delC	p.Leu249Tyrfs*22	Exon 9	HD	Pathogenic	PVS1+PS4 +PM2 + PP4	—	—	—	—	Deletion	PMID: 23799907
17	Familial	c.829C>T	p.Gln277*	Exon 10	PST	Pathogenic	PVS1+PS4 +PM2 +PP4	—	—	—	—	Non-sense	PMID: 16543198
18	Sporadic	c.916 + 1G>A		Intron 10	PST	Pathogenic	PVS1+PS4 +PM2 +PP4	—	—	—	DL (1.0)	Splicing	PMID: 32360764
19	Sporadic	c.949C>T	p.Arg317*	Exon 11	PST	Pathogenic	PVS1+PS4+PS2+PM2 +PP4	—	—	—	—	Non-sense	PMID: 8111379
20	Familial	c.1268A>T	p.X423LextTer14	Exon 13	PST	Pathogenic	PS2+PS4+PM2+PM4+PP4	15.68	—	—	N/E	C-terminal extension	PMID: 11309364
21	Sporadic	c.1268A>T	p.X423LextTer14	Exon 13	PST	Pathogenic	PS2+PS4+PM2 + PM4+PP4	15.68	—	—	N/E	C-terminal extension	PMID: 11309364
22	Sporadic	c.1268A>T	p.X423LextTer14	Exon 13	PST	Pathogenic	PS2+PS4+PM2 + PM4+PP4	15.68	—	—	N/E	C-terminal extension	PMID: 11309364
23	Sporadic	c.1268A>T	p.X423LextTer14	Exon 13	PST	Pathogenic	PS2+PS4+PM2 + PM4+PP4	15.68	—	—	N/E	C-terminal extension	PMID: 11309364

Nucleotide annotation and exon numbering were based on reference sequences NM_000280. The genomic location for the *PAX6* gene was Chr11:31784779-31817961 (GRCh37/hg19).

**FIGURE 4:**
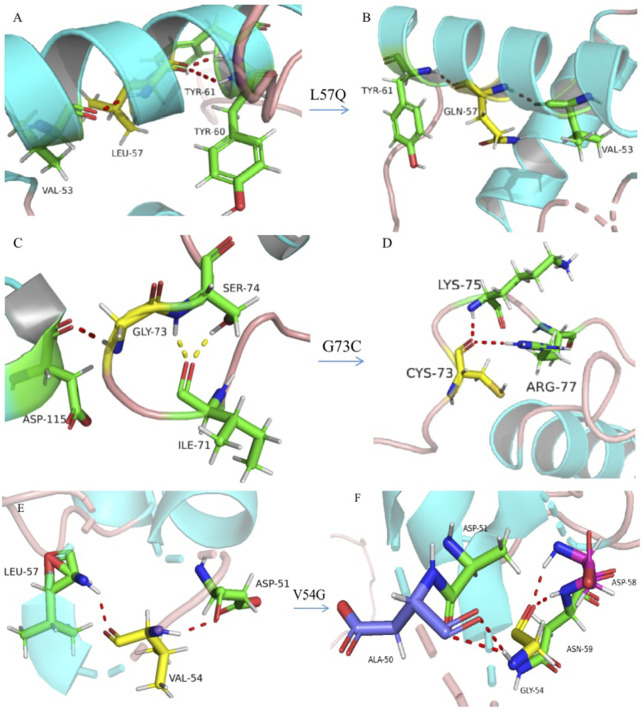
Three-dimensional model construction for missense variations. The model shows that a wild-type non-polar amino acid of leucine (L) was replaced by a polar amino acid of glutamine (Q) at codon 57, which would make the connective hydrogen bands get lost between leucine and tyrosine at codon 60 (**(A)**, wild type; **(B)**, mutant type). Wild-type glycine (G) was replaced by cysteine **(C)** at codon 73, which would make a normal connection between C73 with isoleucine (I) at codon 71, and replaced by an abnormal connection between C73 and arginine (R) at codon 77 (**(C)**, wild type; **(D)**, mutant type). Wild-type valine was replaced by glycine at codon 54, which would make the connective hydrogen band get lost between G54 and leucine at codon 57 and make abnormal connections between G54 and alanine **(A)** at codon 50, aspartic acid **(D)** at codon 58, and asparagine (N) at codon 59 (**(E)**, wild type; **(F)**, mutant type). All aforementioned amino-acid substitutions may damage the stability of the protein structure and function.

Eight novel variations included three missense variations, c.161T>G (p.Val54Gly), c.170T>A (p.Leu57Gln), and c.217G>T (p.Gly73Cys), one non-sense variant of c.688G>T (p.Glu230*), two small deletions of c.357delC (p.Ser119Argfs*5) and c.575_576del (p.Ser192fs), a large deletion of g.20835_23262del, and a novel non-coding variation of c.-128–2A>G at the 5′UTR region of *PAX6* (Table 2; Supplementary Table S2). The novel identified missense variants of c.161T>G (p.Val54Gly), c.170T>A (p.Leu57Gln), and c.217G>T (p.Gly73Cys) were predicted to be deleterious to the protein function and structure using *in silico* analysis by SIFT, PolyPhen-2, and CADD and were further confirmed by three-dimensional model construction using the PyMOL program (Figure 4). The non-sense variant of c.688G>T (p.Glu230*) and two small deletions of c.357delC (p.Ser119Argfs*5) and c.575_576del (p.Ser192fs) were predicted to produce an abnormal mRNA with a premature termination codon (PTC), which would be degraded under the non-sense-mediated decay (NMD) mechanism.

A novel identified variant of c.161T>G (p.Val54Gly) and a known variant of c.1268A>T (p.X423LextTer14) were detected in pedigrees F3 and F12, respectively. Patients in these two families were diagnosed with ASD because they had partial iris absence (iris coloboma) and congenital cataract. In addition, the variation of c.1268A>T (p.X423LextTer14) was also detected independently in three sporadic patients who were diagnosed with aniridia. This variant was predicted to produce a C-terminal extension (CTE). In addition, the known variant of c.141G>A (p.Gln47Gln) is a synonymous mutation that has been reported to affect the splicing process and lead to disease (Dubey et al., 2015).

A large deletion was observed to exist in the *PAX6* gene in pedigree 8 by CNVkit analysis for exons 5–9. Subsequently, qPCR was performed on all exons of *PAX6* to identify the deletions. The copy number ratio for the amplicons of exons 6–8 was found to be half that of exon 6 of *SPATA7* or exon 14 of *TTLL5* (Figure 1), and thus, deletions could be present in exons 6–8. Thus, long-range PCR was performed to detect the deleted regions and the proximal and distal breakpoints of deletion, and a 2,433-bp deletion was found in the patient’s *PAX6* gene beginning from 215 bp proximal to exon 6 to the end of 997 bp distal to exon 8, encompassing exons 6–8. Agarose gel electrophoresis of PCR with a final 2-min extension condition showed that an approximate 0.9-kb band was produced from two affected individuals (the proband and his mother in F8) through long-range PCR amplification but not produced from a normal individual due to a short extension time (Figure 1).

**FIGURE 5:**
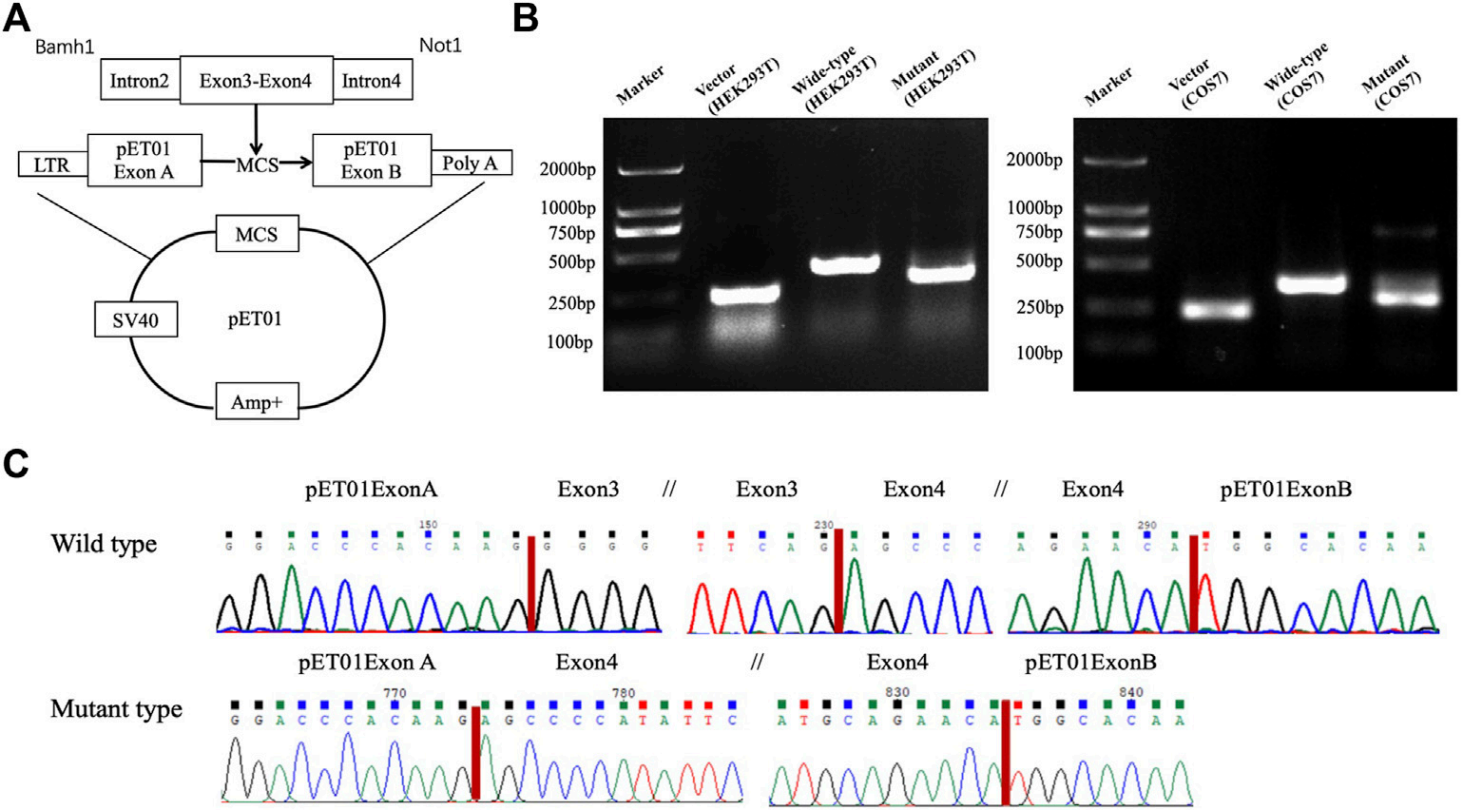
Minigene assay. A skipping of exon 3 was confirmed through the minigene assay using either the HEK293T or the COS7 cell line. Schematic diagram of the pET01 vector and construction of recombinants **(A)**. Agarose gel electrophoresis of RT-PCR products of the wild type and mutant type constructed in the HEK293T and COS7 cell lines **(B)**. Each band indicates the RT-PCR product from an empty vector of pET01 (a positive control) in the second lane from the left, the wild type in the third lane from the left, and the mutant type in the right lane. Sequencing chromatograms **(C)** show a skipping of exon 3 in the mutant type (lower panel), while the correct transcription was kept in the wild type with the entire sequence of exons 3 and 4 and in two exogenous plasmid sequences of exons A and B (upper panel). The break in the sequence is marked with //.

Minigene assay indicated that the variant of c.-128–2A>G altered the splicing process, which resulted in the skipping of exon 3 during transcription. Damage to the splicing process was reproducible in both COS and HEK cell lines, indicating that the experimental results were regardless of the COS7 and HEK293T cell line types used (Figure 5).

All variants and their calculated scores using SIFT, PolyPhen-2, CADD, and SpliceAI, as well as the ACMG classification, are listed in Table 2.

## 4 Discussion

As mentioned in the previous sections, mutations of *PAX6* may lead to various ocular disorders, including congenital aniridia and hypoplasia of the fovea and optic nerve (Souzeau et al., 2018; Lima Cunha et al., 2019). Congenital aniridia can be easily identified based on the typical features of complete iris absence. This disease may develop progressively and present as corneal opacification and vascularization, cataract, or glaucoma (Skeens et al., 2011; Hingorani et al., 2012; Park and Yang, 2018). However, for patients without typical iris absence, genetic testing is helpful in identifying the cause of the disease.

In our study, approximately 64% (29/45) of the patients with the *PAX6* variants showed typical clinical features of congenital aniridia, while other patients with *PAX6* variants showed variable clinical features. The *PAX6* variant types include missense, non-sense, splice-altering, deletion, C-terminal extension, and synonymous variations. With the exception of c.-128-2A>G in the non-coding region of 5′UTR, all variants identified in our study were located in the coding region of the *PAX6* gene.

Recently, the implications of the non-coding *PAX6* variants have been highly concerned with the development of the disease (Plaisancié et al., 2018). The 5′UTR region of the *PAX6* gene plays an important role in keeping mRNA stability and controlling translation (Plaisancié et al., 2018). Variations in the 5′UTR region would disturb the splicing process (Tarilonte et al., 2022); it has been observed in a previous report that the deletion of c.-128-2delA has been found to induce the skipping of exon 3 (Filatova et al., 2021). Our minigene assay results also support the importance of the 5′UTR region based on the fact that the replacement of the invariant A residue at −2 of the acceptor in intron 2 by G also leads to the skipping of exon 3 . To the best of our knowledge, the variant of c.-128-2A>G at the 5′UTR region is reported for the first time in Han Chinese patients with aniridia. Interestingly, the affected individuals with c.-128-2A>G in the F1 pedigree presented with a varied clinical phenotype, even though they carried the same variant.

Patients with the large deletions of g.20835_23262del had a complex phenotype in our cohort. The proband and his mother had a common phenotype of aniridia, microphthalmia, sclerocornea, and congenital cataract, with the exception of bilateral ptosis in his mother. His father had bilateral keratopathy, congenital esotropia, and unilateral ptosis. Microphthalmia is a rare congenital abnormality and belongs to a part of the anophthalmic spectrum, usually associated with coloboma, anterior segment abnormalities, and cataracts. Approximately one-third of microphthalmia cases with coloboma are caused by the defect of the optic fissure closure during the early stage of embryonic development (Plaisancié et al., 2019). Microphthalmia may appear as an isolated ocular condition or as one of the symptoms in some syndromes associated with craniofacial, genital, skeletal, brain, renal, and cardiac abnormalities (Slavotinek, 2019). No more than one-fifth of microphthalmic and anophthalmic cases are explained by pathogenic variants in *SOX2*, *OTX2*, *FOXE3*, *PAX6*, *STRA6*, *ALDH1A3*, *RARB*, *VSX2*, *RAX*, and *BMP4* genes, while a majority of microphthalmic/anophthalmic cases lack a genetic cause at this time (Williamson and FitzPatrick, 2014). Currently, 19 pathogenic variants of *PAX6* have been reported to be associated with microphthalmia, most of which are missense variants. These variants are mainly distributed in the PD, with a few being located in the HD. The large deletion of g.20835_23262del contains the whole exons of 6, 7, and 8 in the *PAX6* gene, whose encoded amino acids are the main part of the PD. This deletion would disturb the interaction between *PAX6* and its target genes, affecting their transcription. There were nine patients who were found to have microphthalmia in our cohort. It is worth noting that many large deletions, even the whole *PAX6* deletion, are not found to be associated with microphthalmia, and on the contrary, some missense mutations could lead to microphthalmia.

Although the spectrum of *PAX6* mutations and their associated clinical phenotypes are well described, there is no clear genotype–phenotype association in most studies (Yokoi et al., 2016; Souzeau et al., 2018; Lima Cunha et al., 2019). Previous studies showed that mutations in PTC and CTE are generally associated with aniridia, while missense mutations are usually related to a milder aniridia phenotype showing a lower frequency of foveal hypoplasia and less severe vision loss (Hingorani et al., 2009; Yahalom et al., 2018). However, in this regard, we did not observe a strict genotype–phenotype correlation in our patients, possibly owing to the small sample size. We found that the phenotype resulting from *PAX6* variants is highly varied in inter- or intra-family members, and the illness severity is more likely to be associated with illness duration, which concurred with the result of a previous longitudinal study on the natural history of aniridia, showing that patients had progressively reduced visual acuity over time. Although we lack statistical analysis, we found the following phenomenon: the longer disease duration leads to visual acuity being impaired severely. For example, in the family with c.-128-2A>G, the proband’s mother had progressively reduced visual acuity from 4/20 to 12/200 during 2 years of cataract occurring in her eyes. In another family with p.M1K, the grandmother of the proband had become blind due to neovascular glaucoma; however, her offspring still have varying degrees of residual vision from hand movement to 2/20.

The variant of c.1268A>T (p.X423LextTer14) is thought to be a mutational hotspot for *PAX6* (Cross et al., 2020). In addition to iris hypoplasia, patients with CTE variants may have variable grades of foveal hypoplasia and aniridia-related keratopathy (ARK). Previous studies showed that whole-gene deletions followed by PTC and CTE variants were associated with the most severe grades of ARK, while the patients with CTE trended toward milder grades of both foveal and iris hypoplasia compared with frameshift and non-sense groups. However, we did not find a correlation between CTE variants and their phenotype in our cohort.

Moreover, the novel variation of c.622C>T (p.Arg208Trp) was detected in a sporadic patient who was initially diagnosed with hypoplasia of the optic nerve and high myopia with a refractive error of less than −5.00 diopters. Two novel variations of c.170T>A (p.Leu57Gln) and c.217G>T (p.Gly73Cys) were detected in two sporadic patients who were diagnosed with congenital foveal hypoplasia because these two patients did not have an iris defect. After *PAX6* variations were detected in these two patients, we re-evaluated their clinical features and found that the patient with c.170T>A (p.Leu57Gln) showed iris ectropion, while another with c.217G>T (p.Gly73Cys) showed corectopia. These examples further illustrate that the phenotype caused by missense variations is complex and difficult to predict.

## 5 Conclusion

We identified eight novel mutations in *PAX6*, which further expanded the mutation spectrum of this gene. The phenotypes caused by the *PAX6* variants are complex. Molecular diagnosis may be helpful in determining the etiology of the corresponding disorders with complicated and occult features.

## Data Availability

The datasets presented in this study can be found in online repositories. The names of the repository/repositories and accession number can be found at: https://www.biosino.org/node/project/detail/OEP003578.
