# Supplementary material for: Diversity of clinical phenotypes in a cohort of Han Chinese patients with PAX6 variants
Source: Front Genet. 2023 Feb 2;14:1011060. doi: 10.3389/fgene.2023.1011060 (PMC9934858; doi:10.3389/fgene.2023.1011060)
Supplement: Supplementary file 1 [file DataSheet1.docx]

Supplemental Tables

Nucleotide annotation and exons numbering were based on reference sequences NM_000280.6 and NG_008679.1.

Table S1 Primer Pairs of PCR reactions in the *PAX6* gene exons

| Exon | Forward primer | Reverse primer | Length(bp) |
| --- | --- | --- | --- |
| 1 | 5’-CCGGCGCTGCTTTGCATAAA-3’ | 5’-AGAGGTGTGGGTGAGGGAAGT-3’ | 298 |
| 2 | 5’-GCCGCTGACAGCCCATTTTA-3’ | 5’-AAGGGGGAGACCTGTCTGAA-3’ | 295 |
| 3 | 5’-TCAGAGAGCCCATGGACGTA-3’ | 5’-TTTTGAGCCCAAAGCAGCCA-3’ | 182 |
| 4 | 5’-AGTTCAGGCCTACCTGATGC-3’ | 5’-CGTCGCGAGTCCCTGTG-3’ | 202 |
| 5 | 5’-TTCCTCTTCCTTCTTCTCCC-3’ | 5’-GGGGTCCATAATTAGCATCG-3’ | 317 |
| 6 | 5’-TGCTGGACAATCAAAACGTAAG-3’ | 5’-ACAGTGGAGAGAGAGGGTGG-3’ | 413 |
| 7 | 5’-TTTTAACGGGTTGAGAGTTGC-3’ | 5’-GAAGCCCTGAGAGGAAATGG-3’ | 362 |
| 8 | 5’-ACCTTGGGAATGTTTTGGTG-3’ | 5’-TTTGAATTACTGGTAGATGAAAAGAAG-3’ | 540 |
| 9 | 5’-AGGTGGGAACCAGTTTGATG-3’ | 5’-CAGAGCATTTAGCAGACTGAACC-3’ | 303 |
| 10 | 5’-TCTGCTAAATGCTCTGCTGC-3’ | 5’-AACATGGAGCCAGATGTGAAG-3’ | 387 |
| 11 | 5’-AATTCCACAACCCACCACAC-3’ | 5’-TGACTGGTCAAGCCAATCAC-3’ | 372 |
| 12 | 5’-CTTGTTGGCAGAGTTCCTCG-3’ | 5’-AGGAGTTAAACACGCCCTCC-3’ | 347 |
| 13 | 5’-AAACCTATAAATTTGTATTCCATG-3’ | 5’-TTTCAAGTCCATTCCTTCCC-3’ | 331 |

Table S2 Primer Pairs of RT-qPCR in exploring a large deletion of exon 6-8 skipping in *PAX6* gene

| Name | Sequence | Chrom_start | Chrom_end | Amplicon length |
| --- | --- | --- | --- | --- |
| PAX6_intron 8.1_qpcr_F | GTGCAACACATTACAAAGAATGG | 31816946 | 31817063 | 118bp |
| PAX6_intron 8.1_qpcr_R | TGACACCTATTACGAGCACAGC | 31816946 | 31817063 | 118bp |
| PAX6_intron 8.2_qpcr_F | CATGGATAAGAATGTGAATCTCCA | 31818802 | 31818921 | 120bp |
| PAX6_intron 8.2_qpcr_R | ATTAAACAAAGTTGCCCGAATCT | 31818802 | 31818921 | 120bp |
| PAX6_intron 8.3_qpcr_F | CAATCCTGTCGCTCTGTAATTG | 31821063 | 31821132 | 70bp |
| PAX6_intron 8.3_qpcr_R | ACGCTTATTCATGTCGCCTTA | 31821063 | 31821132 | 70bp |

Table S3 Primer sequences and product length used in the present study for RT-qPCR

| No. | Internal Reference Gene | Primer Sequence | Product length |
| --- | --- | --- | --- |
| 1 | SPATA7-exon6-qPCR-F | TGAGCTCTGGAGCCCTGTAT | 86bp |
|  | SPATA7-exon6-qPCR-R | GGGTGCTTTCGAAATGACTAAC | 86bp |
| 2 | TTLL5-exon14-qPCR-F | CTGTTAGTGATGCGCCTCTG | 102bp |
|  | TTLL5-exon14-qPCR-R | TGTGAGTCCAGTTCAGAAAATACTAC | 102bp |

Table S4 Phenotypes and genotypes of patients with *PAX6* variations

|  | Case | Mutation | Mutation Type | Gender | Age  (yrs) | BCVA  OD/OS | Aniridia | Keratopathy | Foveal  Hypoplasia | Cataract | Glaucoma | Microp-  hthalmia | Other |
| --- | --- | --- | --- | --- | --- | --- | --- | --- | --- | --- | --- | --- | --- |
|  | F1 |  |  |  |  |  |  |  |  |  |  |  |  |
| 1 | Ⅱ:1 | c.-128-2A>G | Splicing | F | 56 | 0.1/0.1 | C | - | + | + | - | - | - |
| 2 | Ⅲ:2 |  |  | F | 30 | 0.1/0.1 | C | - | + | + | - | - | - |
| 3 | Ⅳ:1 |  |  | M | 6 | 0.2/0.2 | P | - | + | - | - | - | - |
|  | F2 |  |  |  |  |  |  |  |  |  |  |  |  |
| 4 | Ⅱ:3 | c.2T>A  (p.Met1Lys) | Missense | F | 59 | blindness | C | + | + | - | + | - | Atrophy of eyeball |
| 5 | Ⅲ:1 |  |  | F | 34 | blindness | C | + | + | - | + | - | Ectopic lens,  Choroidal  coloboma |
| 6 | Ⅲ:3 |  |  | F | 31 | blindness | C | + | + | - | + | - | Atrophy of optic  nerve |
| 7 | Ⅲ:5 |  |  | M | 27 | 0.1/0.1 | C | - | + | - | - | - | - |
| 8 | Ⅳ:1 |  |  | M | 9 | 0.2/0.2 | C | - | + | - | - | - | - |
| 9 | Ⅳ:3 |  |  | F | 5 | 0.1/0.12 | C | - | + | - | - | - | - |
| 10 | Ⅳ:4 |  |  | F | 8 | 0.2/0.1 | C | - | + | - | - | - | - |
| 11 | Ⅳ:5 |  |  | M | 6 | 0.15/0.2 | C | - | + | - | - | - | - |
| 12 | Ⅳ:6 |  |  | M | 4 | 0.15/0.15 | C | - | + | - | - | - | - |
|  | F3 |  |  |  |  |  |  |  |  |  |  |  |  |
| 13 | Ⅱ:2 | c.161T>G  (p.Val54Gly) | Missense | F | 30 | 0.1/0.1 | P | + | + | + | - | + | - |
| 14 | Ⅲ:1 |  |  | F | 4 | 0.2/0.2 | P | + | + | - | - | + | - |
|  | F4 |  |  |  |  |  |  |  |  |  |  |  |  |
| 15 | Ⅱ:2 | c.170T>G  (p.Leu57Gln) | Missense | F | 41 | 0.2/0.15 | N | - | + | - | - | - | Iris ectropion uvea |
| 16 | Ⅲ:1 |  |  | F | 7 | 0.2/0.2 | N | - | + | - | - | - | - |
|  | F5 |  |  |  |  |  |  |  |  |  |  |  |  |
| 17 | Ⅱ:1 | c.217G>T  (p.Gly73Cys) | Missense | M | 26 | 0.1/0.12 | N | - | + | - | - | - | - |
| 18 | Ⅲ:1 |  |  | F | 2 | NA | N | - | + | - | - | - | Ectopic pupil,  Exotropia |
|  | F6 |  |  |  |  |  |  |  |  |  |  |  |  |
| 19 | Ⅱ:1 | c.357delC  (p.Ser119Rfs*5) | Deletion | M | 28 | 0.05/0.05 | C | - | + | - | - | - | - |
| 20 | Ⅱ:1 |  |  | F | 1 | NA | C | - | + | - | - | - | - |
|  | F7 |  |  |  |  |  |  |  |  |  |  |  |  |
| 21 | Ⅱ:1 | c.357+1G>C | Splicing | M | 31 | 0.1/0.1 | C | - | + | - | - | - | - |
| 22 | Ⅲ:1 |  |  | F | 5 | 0.2/0.2 | C | - | + | - | - | - | - |
|  | F8 |  |  |  |  |  |  |  |  |  |  |  |  |
| 23 | Ⅱ:2 | g.20835_23262del | Deletion | F | 31 | FC/FC | C | + | + | + | - | + | Ptosis |
| 24 | Ⅲ:1 |  |  | F | 6 | 0.15/0.16 | C | + | + | + | - | + | Dysgenesis of the optic disc |
|  | F9 |  |  |  |  |  |  |  |  |  |  |  |  |
| 25 | Ⅱ:1 | c.688G>T  (p.Glu230*) | Nonsense | M | 32 | 0.15/0.1 | C | - | + | - | - | - | Ectopic lens |
| 26 | Ⅲ:1 |  |  | F | 6 | 0.2/0.2 | C | - | + | - | - | - | - |
| 27 | Ⅲ:2 |  |  | F | 4 | 0.2/0.15 | C | - | + | - | - | - | - |
|  | F10 |  |  |  |  |  |  |  |  |  |  |  |  |
| 28 | Ⅱ:1 | c.745delC  (p.Leu249Tyrfs*22) | Deletion | M | 34 | 0.1/0.1 | C | - | + | - | - | - | Ptosis |
| 29 | Ⅲ:1 |  |  | M | 7 | 0.1/0.1 | C | - | + | - | - | - | - |
|  | F11 |  |  |  |  |  |  |  |  |  |  |  |  |
| 30 | Ⅱ:1 | c.829C>T  (p.Gln277*) | Nonsense | F | 68 | 0.05/0.05 | C | - | + | + | - | + | - |
| 31 | Ⅲ:2 |  |  | F | 36 | 0.1/0.1 | C | - | + | + | - | + | - |
| 32 | Ⅳ:1 |  |  | F | 3 | NA | C | - | + | - | - | + | - |
|  | F12 |  |  |  |  |  |  |  |  |  |  |  |  |
| 33 | Ⅱ:3 | c.1268A>T  (p.X423LextTer14) | C-terminal  extension | F | 30 | 0.05/0.05 | P | + | + | + | - | + | High myopia |
| 34 | Ⅲ:1 |  |  | M | 4 | 0.1/0.1 | P | + | + | - | - | + | - |
| 35 | S1 | c.141G>A  (p.Gln47Gln) | Synonymous | F | 5 | 0.1/0.1 | C | + | + | - | - | - | High myopia |
| 36 | S2 | c.622C>T  (p.Arg208Trp) | Missense | M | 4 | 0.2/0.16 | N | - | + | - | - | - | High myopia,  Optic nerve  hypoplasia |
| 37 | S3 | c.916+1G>A | Splicing | M | 1 | NA | C | - | + | - | - | - | - |
| 38 | S4 | c.1268A>T  (p.X423LextTer14) | C-terminal  extension | F | 6 | 0.1/0.1 | C | - | + | - | - | - | - |
| 39 | S5 | c.1268A>T  (p.X423LextTer14) | C-terminal  extension | M | 5 | 0.12/0.1 | C | - | + | - | - | - | - |
| 40 | S6 | c.1268A>T  (p.X423LextTer14) | C-terminal  extension | M | 9 | 0.12/0.12 | N | + | + | - | - | - | - |
| 41 | S7 | c.468G>A  (p.Trp156*) | Nonsense | M | 6 | 0.1/0.1 | C | - | + | - | - | - | - |
| 42 | S8 | c.613C>T  (p.Gln205*) | Nonsense | M | 7 | 0.12/0.2 | C | - | + | - | - | - | - |
| 43 | S9 | c.141+2T>C | Splicing | F | 5 | 0.15/0.2 | C | - | + | - | - | - | - |
| 44 | S10 | c.575_576del  (p.Ser192fs) | Deletion | M | 5 | 0.2/0.2 | C | - | + | - | - | - | Esotropia,  Hyperpresbyopia |
| 45 | S11 | c.949C>T  (p.Arg317*) | Nonsense | M | 6 | 0.1/0.12 | C | + | + | - | - | - | - |


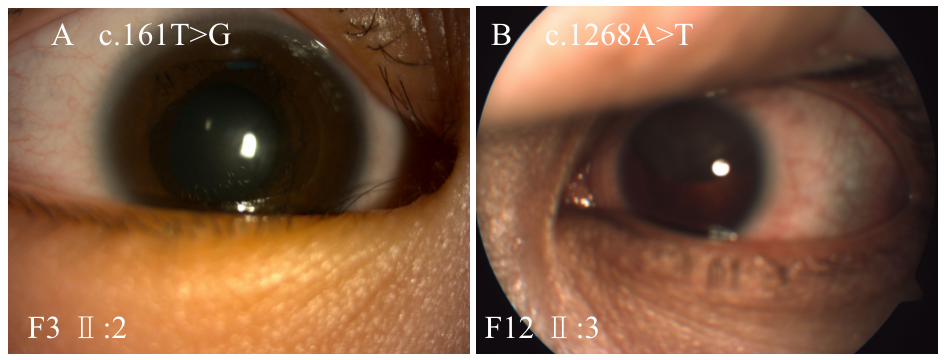


Figure S1. A: Cornea limbal ring lesion, partial absence of iris, mild cataract, and microphthalmia.

B: Cornea limbal ring lesion, partial absence of iris, cataract, and microphthalmia.


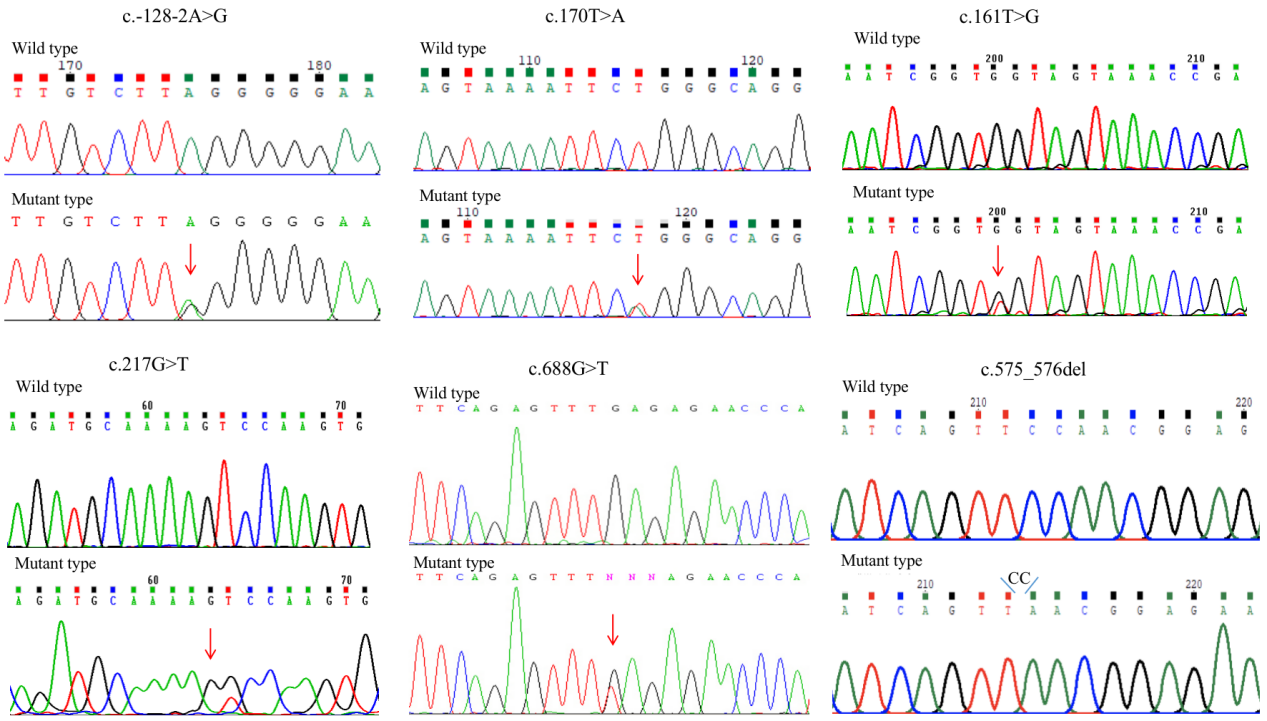


Figure S2. Sequencing chromatograms of eight novel variations identified in the PAX6 gene.
